# Radiomics analysis of pre-treatment [^18^F]FDG PET/CT for patients with metastatic colorectal cancer undergoing palliative systemic treatment

**DOI:** 10.1007/s00259-018-4100-6

**Published:** 2018-08-09

**Authors:** E. J. van Helden, Y. J. L. Vacher, W. N. van Wieringen, F. H. P. van Velden, H. M. W. Verheul, O. S. Hoekstra, R. Boellaard, C. W. Menke-van der Houven van Oordt

**Affiliations:** 1 Cancer Center Amsterdam, Department of Medical Oncology, VUmc, Amsterdam, the Netherlands; 2 Department of Epidemiology and Biostatistics, VU University Medical Center, Amsterdam, the Netherlands; 3 Department of Radiology, Section of Nuclear Medicine, Leiden University Medical Center, Leiden, the Netherlands; 4 Department of Radiology and Nuclear Medicine, VU University Medical Center, Amsterdam, the Netherlands

**Keywords:** [^18^F]FDG PET/CT, Colorectal cancer, Retrospective clinical trial, Radiomics, Tumour heterogeneity, Biomarker

## Abstract

**Background:**

The aim of this study was to assess radiomics features on pre-treatment [^18^F]FDG positron emission tomography (PET) as potential biomarkers for response and survival in patients with metastatic colorectal cancer (mCRC).

**Methods:**

Patients with mCRC underwent [^18^F]FDG PET/computed tomography (CT) prior to first- or third-line palliative systemic treatment. Tumour lesions were semiautomatically delineated and standard uptake value (SUV), metabolically active tumour volume (MATV), total lesion glycolysis (TLG), entropy, area under the curve of the cumulative SUV-volume histogram (AUC-CSH), compactness and sphericity were obtained.

**Results:**

Lesions of 47 patients receiving third-line systemic treatment had higher SUV_max_, SUV_peak_, SUV_mean_, MATV and TLG, and lower AUC-CSH, compactness and sphericity compared to 52 patients receiving first-line systemic treatment. Therefore, first- and third-line groups were evaluated separately. In the first-line group, anatomical changes on CT correlated negatively with TLG (ρ = 0.31) and MATV (ρ = 0.36), and positively with compactness (ρ = −0.27) and sphericity (ρ = −0.27). Patients without benefit had higher mean entropy (*p* = 0.021). Progression-free survival (PFS) and overall survival (OS) were worse with a decreased mean AUC [hazard ratio (HR) 0.86, HR 0.77] and increase in mean MATV (HR 1.15, HR 1.22), sum MATV (HR 1.14, HR 1.19), mean TLG (HR 1.16, HR 1.22) and sum TLG (HT1.12, HR1.18). In the third-line group, AUC-CSH correlated negatively with anatomical change (ρ = 0.21). PFS and OS were worse with an increased mean MATV (HR 1.27, HR 1.68), sum MATV (HR 1.35, HR 2.04), mean TLG (HR 1.29, HR 1.52) and sum TLG (HT 1.27, HR 1.80). SUV_max_ and SUV_peak_ negatively correlated with OS (HR 1.19, HR 1.21). Cluster analysis of the 10 radiomics features demonstrated no complementary value in identifying aggressively growing lesions or patients with impaired survival.

**Conclusion:**

We demonstrated an association between improved clinical outcome and pre-treatment low tumour volume and heterogeneity as well as high sphericity on [^18^F]FDG PET. Future PET imaging research should include radiomics features that incorporate tumour volume and heterogeneity when correlating PET data with clinical outcome.

**Electronic supplementary material:**

The online version of this article (10.1007/s00259-018-4100-6) contains supplementary material, which is available to authorized users.

## Introduction

Currently, [^18^F]fluorodeoxyglucose positron emission tomography/computed tomography ([^18^F]FDG PET/CT) is widely used in the management of colorectal cancer to detect metastases [1]. In recent decades, many studies focused on quantitative assessment of [^18^F]FDG PET and the correlation with clinical outcome. Unfortunately, there is no consensus regarding optimal segmentation methods or quantitative indices to express metabolic characteristics of a tumour lesion. Standard uptake values (SUV) and volume-based indices are most extensively investigated. In patients with colorectal cancer, these measures are demonstrated to be prognostic on pre-treatment [^18^F]FDG PET in neoadjuvant [2, 3] and metastatic settings [4, 5]. However, the corrected tumour activity or metabolically active tumour volume (MATV) are only some of the PET characteristics which can be calculated from PET images. Other structural and textural imaging features might have additional value and can more accurately represent tumour biology. Indices, such as sphericity and compactness describe the shape of a tumour lesion. Heterogeneity can be expressed using entropy, which describes the sum of probability of a voxel grey level within the tumour volume of interest (VOI). Another accepted heterogeneity index is the areas under the curve of the cumulative SUV-volume histogram (AUC-CSH). [6–10]. The interest in tumour heterogeneity is growing, as advances in targeted medicine and knowledge about colorectal cancer biology is increasing. During the course of disease progression, heterogeneity in somatic mutations occur. Heterogeneous tumours grow more aggressively and negatively influence treatment response and patient survival [11, 12]. These genetic alterations influence tumour glucose consumption detected with [^18^F]FDG PET [13]. Using the entire scope of radiomics indices, intralesional tumour heterogeneity in metabolism of [^18^F]FDG can be quantified and differences between lesions can be evaluated. In locally advanced disease, these measures for heterogeneity correlate with recurrence [14] and survival [15]. However, the clinical meaning of these structural and textural indices and added value to the conventional PET units remain unclear for patients with metastatic colorectal cancer (mCRC).

In this study, we retrospectively evaluate the baseline metabolic tumour fingerprint using a comprehensive radiomics panel on baseline [^18^F]FDG PET/CT in relation with clinical outcome for patients with mCRC undergoing palliative systemic therapy. We hypothesized that highly metabolically active and heterogeneous tumour lesions will respond poorly to systemic treatment and have a poor progression-free survival (PFS) and overall survival (OS).

## Methods

### Population

Patient records were evaluated for inclusion if patients had participated in one of seven prospective clinical trials open in the VU University Medical Center in the period from January 2012 until May 2017 (NCT01792934, NCT01998152, NCT02135510, NCT01896856, NCT02117466 and NCT01691391). These studies included patients undergoing first- (capecitabine combined with oxaliplatin with or without bevacizumab) or third-line (cetuximab monotherapy) standard systemic treatment. All patients gave written informed consent to participate in one of the aforementioned studies. The medical ethics commission of the VU University Medical Center approved the retrospective study protocol. Patients with mCRC were included if [^18^F]FDG PET/CT had been performed prior to the start of palliative systemic treatment, with a maximal interval between PET and treatment of 2 months. Patients did not receive any (local) anti-cancer treatment between baseline [^18^F]FDG PET and the start of the evaluated systemic treatment.

### [^18^F]FDG PET/CT

[^18^F]FDG-PET/CT scans were performed and reconstructed according to the EANM guidelines using EARL-accredited PET scanners [16]. Briefly, patients fasted 6 h prior the tracer injection (target serum glucose ≤7 mmol/l). A static whole-body (skull to mid-thigh) PET scan was started 60 min (± 5 min) after injection of [^18^F]FDG (3–4 Mbq/kg), with a scanning time of 2 min per bed position. A low-dose CT (120 kVp, 50 mAs) was acquired prior to the PET scan. All PET data were normalized and corrected for scatter and random events, attenuation and decay.

### Tumour delineation and quantification

PET VOIs were semiautomatically delineated using a threshold of 50% of the SUV_peak_, with correction for local background (SUV ≤4) [17]. All visually identifiable tumour lesions were delineated. Lesions were analysed if SUV_peak_ was higher than background, defined as two times SUV_mean_ of the blood pool (VOI of five voxels in five consecutive planes in the ascending aortic arch) [18].

From each VOI, 10 radiomics indices were calculated. SUV was defined as the activity in a tumour VOI normalised for injected dose and lean body mass. We evaluated three commonly used first-order SUV indices; SUV_max_ (defined by the voxel with the highest activity within VOI), SUV_mean_ (mean activity in the tumour VOI) and SUV_peak_ (SUV_mean_ determined in a 12-mm diameter sphere that was automatically positioned in the VOI to acquire the highest value). The MATV in cm^3^ was determined with a threshold of 50% of the SUV_peak_ (with background correction ≤ SUV 4). Total lesion glycolysis (TLG) was defined as the SUV_mean_ times MATV.

Five textural and structural radiomics indices were evaluated. Entropy expresses heterogeneity in tracer uptake within the tumour VOI on a voxel basis. Entropy consists of the sum of the probability of a certain voxel value. The formula for $entropy\ (Shannon)=-{\sum}_{l=1}^k\left[p(l)\right]\mathit{\log}2\left[p(l)\right]$, *l* is the number of grey levels in the VOI and ranges from 1 to k [19]. The probability of a certain range of grey-level values can be evaluated in steps based on the maximal value k in 64 bins, or in fixed SUV bins (0.25 g/ml) for every VOI (entropy FXD). AUC-CSH is another measure for heterogeneity; it comprises the AUC of the histogram of the % of total tumour volume above % threshold of SUV_max_, calculated with the Riemann sum using the trapezoidal rule [20]. This results in a low AUC-CSH for heterogeneous lesions. Thus, homogeneous tumours would have higher entropy and AUC-CSH compared to heterogeneous tumours.

Sphericity is a measure to describe the sphere-like shape of the VOI. $Sphericity=\frac{{\left(36\uppi {V}^2\right)}^{1/3}}{\mathrm{A}}$. V is defined as volume and A as surface area of the VOI. A is defined as the sum of length times width of every plan in the VOI. Much like sphericity, compactness describes the deviation of the VOI from a perfect sphere. $Compactness=\frac{\mathrm{V}}{\uppi^{1/2}{\mathrm{A}}^{3/2}}$ [21]. Thus, spherical tumours would have higher sphericity and compactness compared to aspherical tumours.

The correlation between clinical outcome measures and all PET features were evaluated for lesions with a metabolic volume ≥ 4.2 mL, as these lesions are less affected by partial volume effects [22]. For the analysis at a patient level, the mean of all metastases was calculated for all PET features. To assess total tumour bulk per patient, the sum of MATV and TLG of all lesions was evaluated (independent of volume).

### Clinical outcome

In this study, four clinical outcome measures were evaluated: anatomical change on CT per lesion, treatment benefit, PFS and OS. Treatment benefit was defined as stable disease or response versus progressive disease (PD) on first-evaluation CT scan (2–3 months) according to RECIST v1.1. Briefly, RECIST v1.1 response evaluation entails evaluation of maximally 5 lesions [≤2 per organ, lesion diameter ≥ 10 mm (long axis) or ≥ 15 mm (short axis) for lymph nodes]. PD is defined as ≥20% increase and non-PD as <20% increase of the sum of diameters. Additionally, all quantified tumour lesions (above background) were measured on the baseline and first-evaluation CT, with the exception of non-measurable lesions (e.g. bone lesions or pleural carcinomatosis). PFS and OS were defined as the period starting from the date of the first evaluated treatment cycle to the date of PD or death, respectively. Follow-up was continued until the first of August 2017.

### Statistical analysis

All statistical analyses were performed using IBM SPSS version 22, with the exception of the cluster analysis, which was performed using R version 3.2.3. Benefit and survival analysis were performed separately for each treatment line, as first-line treatment is expected to lead to better response rates and longer survival compared to third-line treatment. The normality of PET features was evaluated using histograms. Correlations between PET features and change on CT were investigated using a Pearson’s correlation for normally distributed data, i.e. SUV_max_, SUV_peak_, SUV_mean_, AUC-CSH, entropy, entropy FXD, compactness and sphericity. Spearman’s rho was used in skewed data, i.e. MATV and TLG. For linear correlations, explained variance was defined as the square of the correlation coefficient. Radiomics features that demonstrated a significant univariate linear correlation with change on CT were evaluated using linear mixed-effects models to correct for clustering within a patient (skewed data was log transformed). A random intercept with one fixed factor (non-random slope) was used, with restricted maximum likelihood and unstructured covariance type. To compare differences in PET features for a patient with and without treatment benefit, RAS/BRAF mutations and sidedness of primary tumour, independent *t* tests were used for normally distributed values, i.e. SUV_max_, SUV_peak_, SUV_mean_, AUC-CSH, entropy, entropy FXD, compactness and sphericity. Mann-Whitney U tests were used in skewed data, i.e. MATV and TLG. Additionally, using a receiver operator characteristic (ROC) curve, the area under the ROC was calculated to give insight into the sensitivity and specificity of PET features.

For the survival analysis, patients without progression and patients that are still alive were censored at August 1st 2017. Univariate survival analysis was done using Cox regression to evaluate potential correlation between baseline PET features and either OS or PFS. To calculate a meaningful hazard ratios (HRs), continuous variables were binned in 10% percentiles. Multivariate analyses was performed using Cox regression with the "Enter" method. After continuous correlation of the PET features, data were dichotomized based on the 50th percentile and evaluated with Kaplan–Meier curves (log rank).

The 10 radiomics features were clustered using the partitioning around medoids and hierarchical clustering method. For both methods, the number of clusters was selected by means of consensus clustering, which selects the most stable clustering. As a way of technical validation, found clusters were visualized in a principal component plot.

## Results

### Patient characteristics

**Fig. 1:**
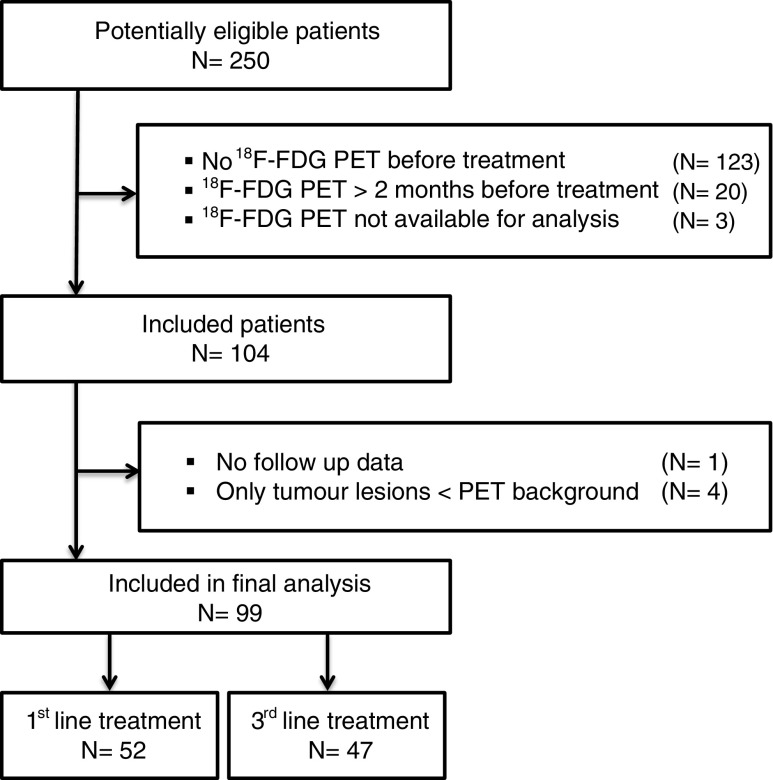
Flow chart of patient inclusion

Of the 250 patients included in aforementioned studies, 104 were eligible for this analysis. One of these patients was lost in follow-up and four had no evaluable lesions on FDG PET. Thus, 99 patients were included in the final analysis (Fig. 1). Fifty-two were treated in first-line setting (capecitabine combined with oxaliplatin with or without bevacizumab); the remaining 47 were treated with third-line cetuximab monotherapy. Patient characteristics are described in Table 1.

**Table 1:** Patient characteristics

	Total	First-line treatment	Third-line treatment
No. patients	99	52	47
Median age (min–max)	64.7 (22–84)	65.8 (22–84)	63.4 (49–82)
Male gender	65.7%	59.6%	72.3%
Primary tumour			
Right-sided	27.3%	30.8%	23.4%
Left-sided	72.7%	69.2%	76.%
Mutation status			
RAS	15.2%	25.9%	0%
RAS wild-type	50.5%	9.6%	100%
Unknown RAS status	34.3%	64.5%	0%
BRAF mutated	4%	0%	8.5%
BRAF wildtype	55.6%	32.7%	80.9%
Unknown BRAF status	40.4%	67.3%	10.6%
Location tumour depositions			
Liver metastases	56.2%	51.1%	49.7%
Lymph nodes	14.1%	17.6%	22.1%
Primary tumour	7.9%	9.6%	2.6%
Other	21.8%	21.7%	25.6%
Evaluated treatment			
CAPOX-B		63.5%	–
CAPOX		28.8%	–
Capecitabine-B		5.8%	–
Capecitabine		1.9%	–
Cetuximab		–	100%
Local treatment		9%	–
Treatment benefit			
No	21.2%	13.5%	29.8%
Yes	77.8%	84.6%	70.2%
Unknown	1%	1.9%	0%
Time PET treatment (mean days; range)	12 (61)	30 (61)	6 (29)
Time CT treatment (mean days; range)	18 (57)	16 (56)	9 (42)
RECIST v1.1			
PD	22.2%	11.5%	34.0%
SD	50.5%	50.0%	51.0%
PR	24.2%	32.7%	14.9%
Unknown	3.0%	5.8%	0%
PD at time of analysis	85.9%	76.9%	95.7%
Alive at time of analysis	32.3%	38.5%	25.5%
Median PFS in months (min–max)	6.1 (0.8–31.6)	10.5 (0.8–31.6)	4.3 (1.1–21.4)
Median OS in months (min–max)	12.9 (1.1–35.2)	16.1 (1.1–35.2)	9.0 (1.5–27.6)

In these 99 patients, 584 lesions were quantified on [^18^F]FDG PET. Of these lesions, 354 had an MATV of ≥4.2 mL and were included in the analyses. On baseline CT, lesions were smaller (32 versus 41 mm, *p* < 0.001) and tumour shrinkage at first CT evaluation was greater in the first-line group than in the third-line group (mean −21.9% versus −5.3%, *p* = 0.004). On [^18^F]FDG PET, tumour lesions of patients in the first-line group had lower SUV_max_ [mean 6.8 (SD 3.1) versus 7.8 (SD 2.9), *p* = 0.004], SUV_peak_ [mean 5.4 (SD 2.4) versus 6.4 (SD 2.3), *p* < 0.001], SUV_mean_ [mean 4.4 (SD 1.8) versus 4.9 (1.7), *p* = 0.013], MATV [median 9.8 (110.4) versus 16.6 (SD 110), *p* < 0.001], TLG [median 39.3 (SD 672.3) versus 83.3 (SD 589), *p* < 0.001] compared to tumour lesions of patients in the third-line group. Compactness [mean 0.04 (SD 0.01) versus 0.03 (SD 0.013), *p* < 0.001]), sphericity [mean 0.86 (SD 0.16) versus 0.70 (SD 0.2), *p* < 0.001) and AUC-CSH [mean 0.74 (SD 0.05) versus 0.69 (SD 0.13), *p* < 0.001] were higher in the first-line group. Entropy [mean 5.3 (SD 0.21) versus 5.3 (SD 0.31) *p* = 0.4] and entropy FXD [mean 3.6 (SD 0.68) versus 3.6 (SD 0.91) *p* = 0.9] was not different between the first-line and third-line groups.

### First-line treatment group

#### Analysis on a lesion level

In the first-line treatment group, 70% of 136 lesions were measurable on CT. There was a positive but weak correlation with percentage change on CT and MATV (*p* < 0.001, ρ = 0.36), TLG (*p* = 0.002, ρ = 0.31), compactness and sphericity (*p* = 0.009, ρ = −0.27 for both). Correction for clustering of lesions within patients MATV (*p* = 0.007, estimate log MATV 16.1, 95% CI 4.5–27.6) and TLG (*p* = 0.01, estimate log TLG 13.2, 95% CI 3.3–23.2) remained correlated with change on CT; compactness (*p* = 0.23) and sphericity (*p* = 0.23) did not.

SUV_max_ (*p* = 0.4), SUV_peak_ (*p* = 0.6), SUV_mean_ (*p* = 0.45), MATV (*p* = 0.38), TLG (*p* = 0.56) and entropy (*p* = 0.51) were not different between different organ sites of metastases. Yet, compactness (*p* = 0.03), sphericity (*p* = 0.03) and AUC-CSH (*p* = 0.04) were significantly different (supplementary data 1).

#### Analysis on a patient level

Patients without treatment benefit had a significantly higher mean entropy compared to patients with benefit (5.38 versus 5.27, *p* = 0.04 respectively, Table 2). The ROC curve for mean entropy demonstrates that it is a fairly good predictor for treatment benefit with an area under the ROC of 0.74 (95% CI 0.52–0.97, supplemental figure 1).

**Table 2:** Radiomics versus clinical outcome in first-line treatment

First-line treatment group													
	Treatment benefit					Progression-free survival				Overall survival			
	Yes		No		p		95% CI		p		95% CI		p
	Mean	SD	Mean	SD		HR	Lower	Upper		HR	Lower	Upper	
Mean SUV_max_	7.28	3.67	6.90	2.33	0.80	0.98	0.86	1.10	0.69	1.06	0.93	1.20	0.38
Mean SUV_peak_	5.56	2.67	5.55	1.82	0.99	1.03	0.91	1.16	0.69	1.08	0.96	1.23	0.21
Mean SUV_mean_	4.65	2.08	4.52	1.38	0.55	0.98	0.87	1.10	0.67	1.06	0.94	1.21	0.33
Mean compactness	0.04	0.01	0.04	0.01	0.78	0.94	0.84	1.07	0.35	0.87	0.76	1.00	0.06
Mean sphericity	0.87	0.11	0.88	0.09	0.80	0.94	0.84	1.07	0.35	0.87	0.76	1.00	0.06
Mean AUC-CSH	0.74	0.04	0.73	0.02	0.55	0.86	0.76	0.97	0.02*	0.77	0.66	0.89	<0.01*
Mean entropy	5.27	0.14	5.38	0.12	0.04*	1.10	0.97	1.25	0.14	1.01	0.89	1.16	0.84
Mean entropy FXD	3.64	0.67	3.72	0.52	0.77	1.02	0.90	1.16	0.75	1.09	0.95	1.25	0.20
Mean MATV	12.05	25.68	17.05	79.75	0.06	1.15	1.03	1.28	0.01*	1.22	1.07	1.40	<0.01*
SUM MATV	25.41	199.93	51.46	242.16	0.14	1.14	1.02	1.29	0.02*	1.19	1.04	1.36	0.01*
Mean TLG	44.54	183.14	83.66	482.52	0.07	1.16	1.03	1.30	0.02*	1.22	1.06	1.41	0.01*
SUM TLG	97.67	1178.42	200.81	1461.67	0.25	1.12	1.00	1.26	0.05*	1.18	1.03	1.35	0.02*

PFS was positively correlated with mean AUC-CSH (*p* = 0.02, HR 0.86, 95% CI 0.76–0.97) and negatively correlated with mean MATV (*p* = 0.01, HR 1.15, 95% CI 1.03–1.28), sum MATV (p = 0.02, HR 1.14, 95% CI 1.02–1.29), mean TLG (p = 0.02, HR 1.16, 95% CI 1.03–1.30) and sum TLG (*p* = 0.05, HR 1.12, 95% CI 1.00–1.26, Table 2). With multivariate analyses, corrected for performance status, sidedness and RAS or BRAF mutation status, none of the radiomics features correlated with PFS.

**Fig. 2:**
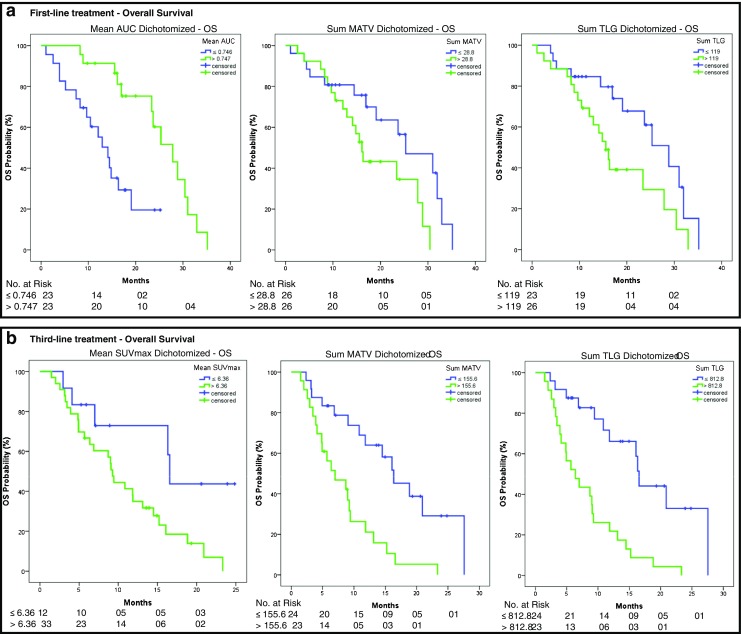
**a** Differences in survival for patients undergoing first-line treatment based on dichotomized data using the 50th percentile of mean AUC-CSH, sum MATV and sum TLG. **b** Differences in survival for patients undergoing third-line treatment based on dichotomized data mean SUVmax, sum MATV and TLG

Similar to PFS, OS was positively correlated with mean AUC-CSH (*p* < 0.01, HR 0.77, 95% CI 0.66–0.89) and negatively correlated with mean MATV (*p *< 0.01, HR 1.22, 95% CI 1.07–1.40), sum MATV (*p* = 0.01, HR 1.19, 95% CI 1.04–1.36), mean TLG (*p *= 0.01, HR 1.22, 95% CI 1.06–1.41) and sum TLG (*p* = 0.02, HR 1.18, 95% CI 1.03–1.35, Table 2). Dichotomization based on the 50th percentile showed a significantly shorter OS for patients with a low AUC-CSH (median 14.1 versus 27.9 months, *p* = 0.001), high sum MATV (median 16.1 versus 25.3 months, *p* = 0.036) and high sum TLG (median 15.6 versus 28.8 months, *p* = 0.027, Fig. 2a). With multivariate analyses, corrected for performance status, number of metastases, sidedness and RAS or BRAF mutation status, AUC-CSH (*p* = 0.016, HR 0.64, 95% CI 0.45–0.92) and sum MATV (*p* = 0.048, HR 2.63, 95% CI 1.01–6.87) correlated with OS. Mean and sum TLG and mean MATV did not (*p* = 0.34, *p* = 0.41 and *p* = 0.25, respectively).

Patients with right-sided CRC had significantly higher mean MATV (median 12.5 versus 17.8, *p* = 0.049), sum MATV (median 24.3 versus 49.4, *p* = 0.043) and sum TLG (median 106.3 versus 382.7, *p* = 0.031). There was no significant relation between radiomics features and BRAF and RAS mutation status (supplemental table 1A).

### Third-line treatment group

#### Analysis on a lesion level

**Fig. 3:**
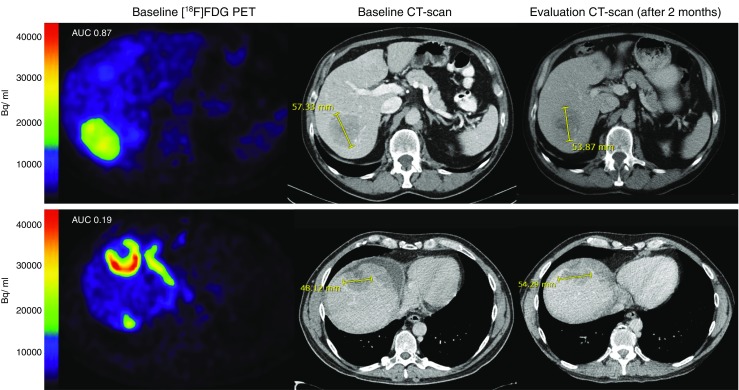
AUC-CSH and response on CT for two patents undergoing third-line cetuximab monotherapy

In the third-line treatment group, 82% of 218 lesions were measurable on CT. Heterogeneity expressed as AUC-CSH was positively but weakly correlated with percentage change on CT (ρ = 0.21, *p* = 0.005, Fig. 3). Yet, after correction for clustering within patients, AUC-CSH was not correlated with change on CT (*p* = 0.35).

MATV (*p* = 41), TLG (*p* = 0.20), compactness (*p* = 0.22), sphericity (*p *= 0.22) and AUC (*p* = 0.44) were not different between different organ sites of metastases. Yet, SUV_max_ (*p* < 0.01), SUV_peak_ (*p *< 0.01), SUV_mean_, (*p *< 0.01) and entropy (*p *< 0.01) were significantly different (supplementary data 1).

#### Analysis on a patient level

There were no significant differences in radiomics features for patients with and without treatment benefit (Table 3).

**Table 3:** Radiomics versus clinical outcome in third-line treatment

Third-line treatment group													
	Treatment benefit					Progression-free survival				Overall survival			
	Yes		No		p	HR	95% CI		p	HR	95% CI		p
	Mean	SD	Mean	SD			Lower	Upper			Lower	Upper	
Mean SUV_max_	7.71	2.30	9.27	4.00	0.10	1.11	0.96	1.27	0.16	1.19	1.01	1.41	0.03*
Mean SUV_peak_	6.42	1.84	7.36	3.00	0.20	1.11	0.96	1.29	0.15	1.21	1.01	1.45	0.04*
Mean SUV_mean_	4.81	1.45	5.53	1.76	0.16	1.07	0.95	1.20	0.27	1.11	0.97	1.28	0.15
Mean compactness	0.03	0.01	0.03	0.01	0.12	0.90	0.78	1.04	0.16	0.88	0.74	1.05	0.15
Mean sphericity	0.71	0.16	0.61	0.21	0.10	0.90	0.78	1.04	0.17	0.88	0.74	1.05	0.15
Mean AUC-CSH	0.68	0.10	0.71	0.05	0.28	0.92	0.81	1.03	0.16	0.93	0.82	1.05	0.23
Mean entropy	5.35	0.17	5.31	0.22	0.48	1.00	0.91	1.10	0.98	0.96	0.86	1.07	0.45
Mean entropy FXD	3.61	0.63	4.00	0.64	0.07	1.03	0.91	1.16	0.69	1.07	0.94	1.22	0.31
Mean MATV	32.54	95.33	30.34	90.98	0.81	1.27	1.05	1.54	0.02*	1.68	1.20	2.37	<0.01*
SUM MATV	160.33	289.30	156.77	160.88	0.71	1.35	1.09	1.68	0.01*	2.04	1.36	3.07	<0.01*
Mean TLG	187.26	471.51	175.13	615.03	0.71	1.29	1.06	1.56	0.01*	1.54	1.15	2.05	<0.01*
SUM TLG	773.33	1540.94	841.75	812.74	0.51	1.27	1.06	1.53	0.01*	1.80	1.24	2.61	<0.01*

PFS was correlated with mean MATV (*p* = 0.02, HR 1.27, 95% CI 1.05–1.54), sum MATV (*p* = 0.01, HR 1.35, 95% CI 1.09–1.68), mean TLG (*p *= 0.01, HR 1.29, 95% CI 1.06–1.56) and sum TLG (*p *= 0.01, HR 1.27, 95% CI 1.06–1.53, Table 3). With multivariate analyses corrected for performance status, number of metastases, sidedness and RAS or BRAF mutation status, mean MATV (*p* = 0.03, HR 1.35, 95% CI 1.02–1.78), sum MATV (*p *= 0.01, HR 1.43, 95% CI 1.08–1.91), mean TLG (*p* = 0.016, HR 1.45 95% CI 1.07–1.97) and sum TLG (*p *= 0.03, HR 1.35, 95% CI 1.02–1.79) remained correlated with PFS.

OS was negatively correlated with mean MATV (*p* < 0.01, HR 1.68, 95% CI 1.20–2.37), sum MATV (*p *< 0.01, HR 2.04, 95% CI 1.36–3.07), mean TLG (*p *< 0.01, HR 1.54, 95% CI 1.15–2.05) and sum TLG (*p *< 0.01, HR 1.80, 95% CI 1.24–2.61). Additionally, OS was negatively correlated with mean SUV_max_ (*p *= 0.03, HR 1.19, 95% CI 1.01–1.41) and SUV_peak_ (*p* = 0.04, HR 1.21, 95% CI 1.01–1.45, Table 3). With multivariate analyses, mean MATV (*p *< 0.01, HR 2.41, 95% CI 1.38–4.25), sum MATV (*p *< 0.01, HR 2.47, 95% CI 1.45–4.18), mean TLG (*p *< 0.01, HR 1.70, 95% CI 1.15–2.51) and sum TLG (*p *< 0.01, HR 1.72, 95% CI 1.16–2.54) remained correlated with OS. Mean SUV_max_ (*p* = 0.42) and mean SUV_peak_ (*p* = 0.25) did not correlate to OS in multivariate analysis.

Dichotomized data based on the 50th percentile of mean MATV (*p *= 0.04, 16.1 versus 9.1 months), sum MATV (*p* = 0.001, median 16.3 versus 7.0 months), mean TLG (*p* = 0.033, 16.1 versus 9.3 months) and TLG (*p* < 0.001, 16.3 versus 6.4 months) resulted in a significantly different OS between groups (Fig. 2b).

There were no significant differences in the radiomics indices for BRAF or RAS mutated tumours. Patients with right-sided disease had less spherical (compactness *p *= 0.03, mean 0.024 versus 0.033; sphericity *p* = 0.02, mean 0.56 versus 0.71) and less heterogeneous disease (entropy FXD *p* = 0.029, mean 4.12 versus 3.62) compared to left-sided disease (supplementary Table 1B).

### Cluster analysis

#### First-line treatment group

**Fig. 4:**
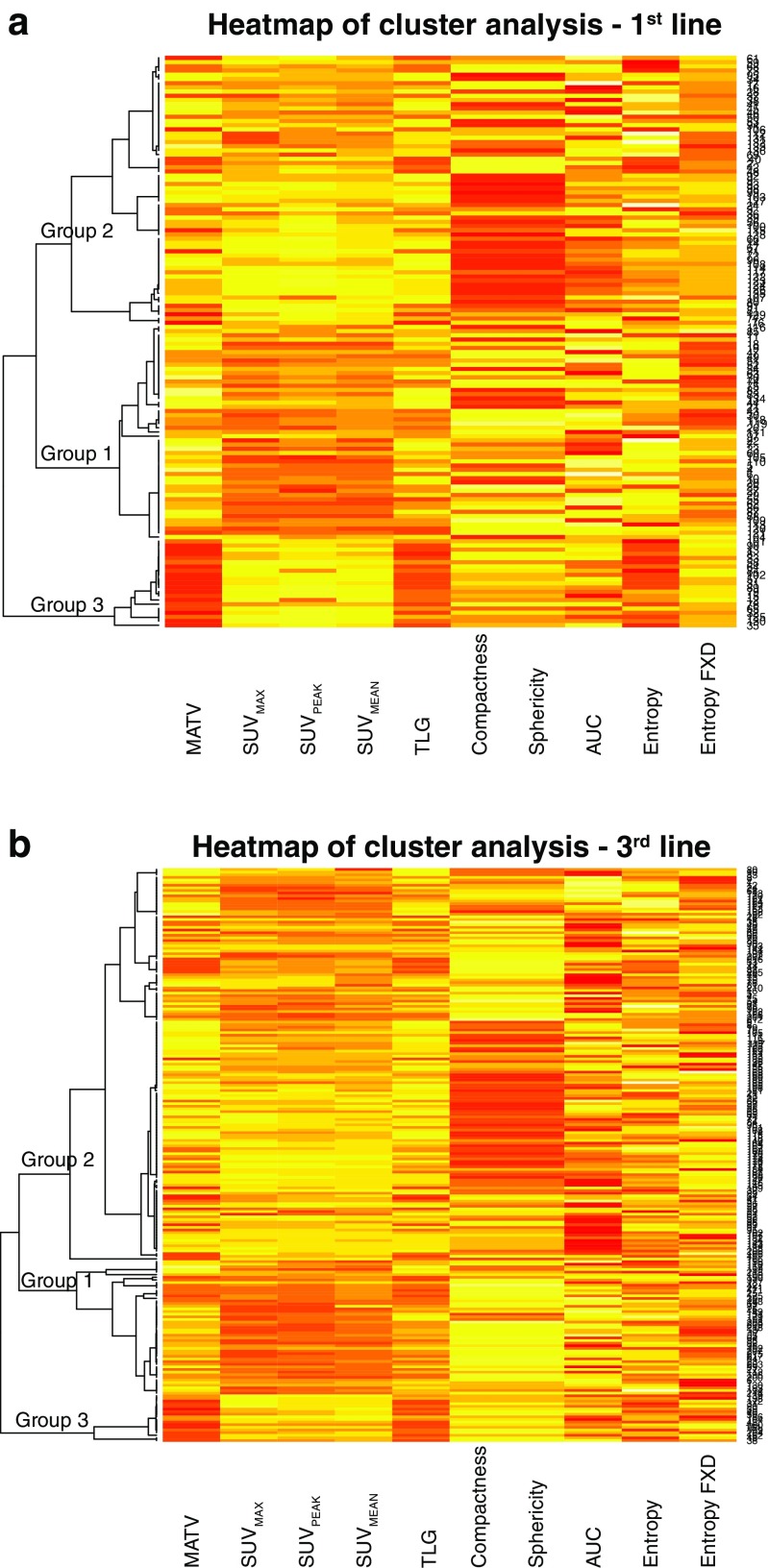
**a** A heatmap of the cluster analysis results of 10 PET characteristics per lesion in the first-line treatment group demonstrates 3 cluster groups. **b** Here, the heatmap of the three cluster groups for the third-line treatment group is illustrated

To evaluate potential complementary predictive and prognostic value, 10 radiomics features were combined in a cluster analysis. Three cluster groups were identified (Fig. 4a); concordance with an alternative cluster analysis was 78%. A consensus-clustering graph demonstrates repeatability of clustering within different subsets of our data set (supplemental figure 2A) and a principal component analysis demonstrates the separation in the 3 groups, based on a summary of the 10 PET features (supplemental figure 2B).

There were no significant differences between the three cluster groups and percentage change on first CT evaluation (*p* = 0.65), PFS (*p* = 0.16) and OS (*p* = 0.37).

#### Third-line treatment group

With the two cluster methods, concordance was 86.1%; the consensus clustering graph and a principal component analysis are shown in supplemental figure 4A and 4B.

**Fig. 5:**
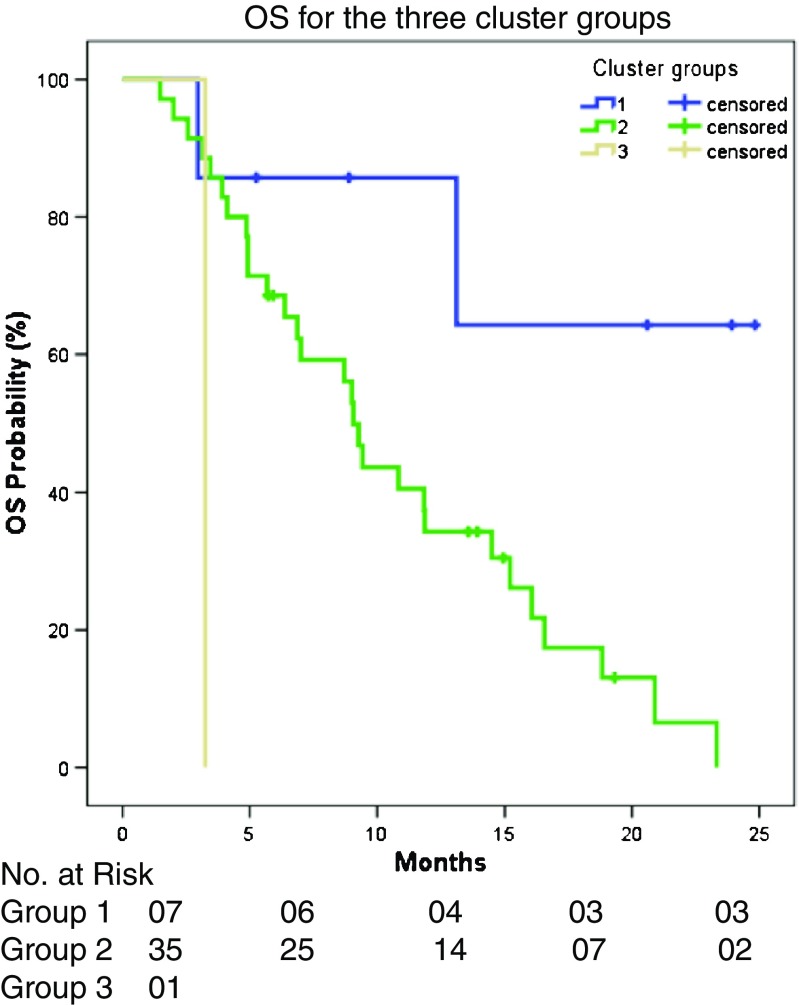
OS for clusters 1, 2 and 3 in the third-line treatment group

As in first-line, the percentage change on first CT evaluation and PFS were not significantly different between the three cluster groups (*p* = 0.22 and *p* = 0.09). However, OS was different, with significantly poorer survival for group 2 versus group 1 (*p* = 0.03, HR 5.03, 95% CI 1.17–21.7, Fig. 5).

#### Association between PET features

Almost all PET features were associated with each other (supplemental table 2). All SUV-based measurements (r^2^ 95–97%) and compactness and sphericity (r^2^ 99.4%) were highly correlated. However, neither compactness nor sphericity were correlated with entropy and entropy FXD. Furthermore, there was no association between AUC-CSH and SUV_mean_ or entropy FXD.

## Discussion

In this study, we demonstrated a relation between total tumour volume, shape and heterogeneity of tracer uptake on pre-treatment [^18^F]FDG PET and clinical outcome. TLG, MATV, compactness and sphericity correlated with anatomical change in the first-line group and AUC-CSH in the third-line group. Yet, with correction for clustering, only TLG and MATV remained correlated with anatomical change. Mean entropy correlated with treatment benefit. For both treatment lines, higher tumour bulk (mean and sum MATV and TLG) was negatively correlated to PFS and OS. Thus, tumour heterogeneity and tumour bulk influences survival despite palliative systemic treatment.

First-order SUV features are most frequently used in clinical care and in studies. In our study, pre-treatment SUV_max_ and SUV_peak_ correlated with OS in the third-line group; however, this was not significant in the multivariate analysis. SUV_max_, SUV_peak_ and SUV_mean_ did not correlate with any other clinical outcome measures. We demonstrate that radiomics, which incorporates biological features such as tumour volume, shape and heterogeneity, correlates better with response and survival data. For future studies, it is important to include these measures when associating PET data with clinical outcome.

Our data indicates that MATV and TLG are promising prognostic features, as these features correlate with PFS and OS in uni- and multivariate regression for patients starting first- and third-line treatment. In literature, a correlation between pre-treatment TLG and OS has been described for both curative [3, 23, 24] and palliative [25, 26] regimens. In line with the literature, our data suggests that a highly metabolically active tumour bulk before start of palliative systemic treatment is a poor prognostic factor, both for patients starting first-line treatment and heavily pre-treated patients. The correlation between tumour load and survival is known for ovarian cancer [27], and is the main rational for current studies investigating debulking of patients with metastasized colorectal cancer as palliative treatment, such as the ORCHESTRA study (NCT01792934). This study evaluates if reduction of tumour load improves OS of patients with mCRC.

Chromosomal tumour instability is a known hallmark of tumour aggressiveness and associated impaired survival. mCRC is heterogeneous in its genetic alterations [11, 28] and palliative systemic treatment increases heterogeneity over time. It has been reported that these genetic alterations influence tumour uptake on [^18^F]FDG PET/CT [13, 29]. With the radiomics features, such as entropy and AUC-CSH, heterogeneity in voxel uptake within lesions can be assessed [21]. Entropy evaluates the sum of the probability of a certain voxel value in the tumour VOI [19]. The AUC-CSH is calculated based on the histogram of the voxel values in the tumour VOI [20]. Homogeneous [^18^F]FDG uptake would result in a high entropy and AUC-CSH. AUC-CSH was significantly higher for first-line lesions compared to third-line lesions, indicating that third-line lesions are more heterogeneous. Additionally, AUC-CSH was positively correlated with change on CT. In line with our data, it has been reported that heterogeneous [^18^F]FDG uptake in colorectal cancer is correlated with poor clinical outcome, such as recurrence and survival [14, 15, 30].

Entropy and entropy FXD were the only two radiomics that were not different between first- and third-line treatment. In contrast to previous data, entropy was higher in patients whom did not respond to first-line treatment and was higher in lesions originating from cancer in the right hemicolon in the third-line cohort. Biologically, it is not logical that homogeneous tumours respond less. However, this result might be due to the semiautomatic delineation. The cut-off for lesion delineation was set at 50% of the SUV_peak_, such that tumours with an intense focal uptake will have a higher cut-off and this could result in less heterogeneity.

Sidedness of the primary tumour is a surrogate prognostic biomarker, and lesions originating from right-sided primary tumours harbour genetic alterations associated with resistance to anti-EGFR therapy [31, 32]. Indeed, using the [^18^F]FDG PET data, we found poorer metabolic features for patients with right-sided disease, such as a higher tumour bulk in patients in the first-line group and less spherical disease in patients in the third-line group. Another potentially meaningful radiomics feature is the shape of a lesion. Aspherical tumour growth has shown to be a poor prognostic marker for breast and lung cancer [33, 34]. In this study, compactness and sphericity were evaluated. However, as both values have a near-perfect concordance, evaluating both would be redundant. In the first-line treatment group, aspherical lesions grow faster on CT.

Using a cluster analysis, we evaluated if certain clusters of PET features give complementary value in characterizing particularly indolent or aggressively growing lesions. The three clusters of lesions did not have differences in anatomical changes on CT. Per patient there were no differences in treatment benefit and PFS. We identified one cluster group with significantly longer survival after third-line treatment. However, the cluster groups had no additional predictive value compared to the individual units. An explanation for the low complementary value of the 10 PET units could be the high correlation among these PET units [35].

This study is limited by the number of included patients. Therefore, we only selected and evaluated 10 out of hundreds of radiomics features, based on previous studies and potential clinical interest [2–12, 14, 15]. Moreover, it is of utmost importance to explore the robustness of these features, assess redundancy and study their dependence on image quality and reconstruction settings as well as image processing steps. It is of interest to mention that a recent initiative, i.e. the Imaging Biomarker Standardisation Initiative (IBSI), is a first important step towards standardization of radiomics features [36].

In conclusion, these data demonstrates that baseline tumour heterogeneity, asphericity and high tumour volume on [^18^F]FDG PET is correlated with impaired benefit and survival despite palliative systemic treatment. Future PET imaging research should not only focus on first-order SUV measures, but also evaluate radiomics that incorporate tumour volume and heterogeneity.

## Electronic supplementary material

ESM 1 (PDF 826 kb)
