# Supplementary material for: Radiomics analysis of pre-treatment [18F]FDG PET/CT for patients with metastatic colorectal cancer undergoing palliative systemic treatment
Source: Eur J Nucl Med Mol Imaging. 2018 Aug 9;45(13):2307–17. doi: 10.1007/s00259-018-4100-6 (PMC6208805; doi:10.1007/s00259-018-4100-6)
Supplement: Supplementary file 1 — (PDF 826 kb) [file 259_2018_4100_MOESM1_ESM.pdf]

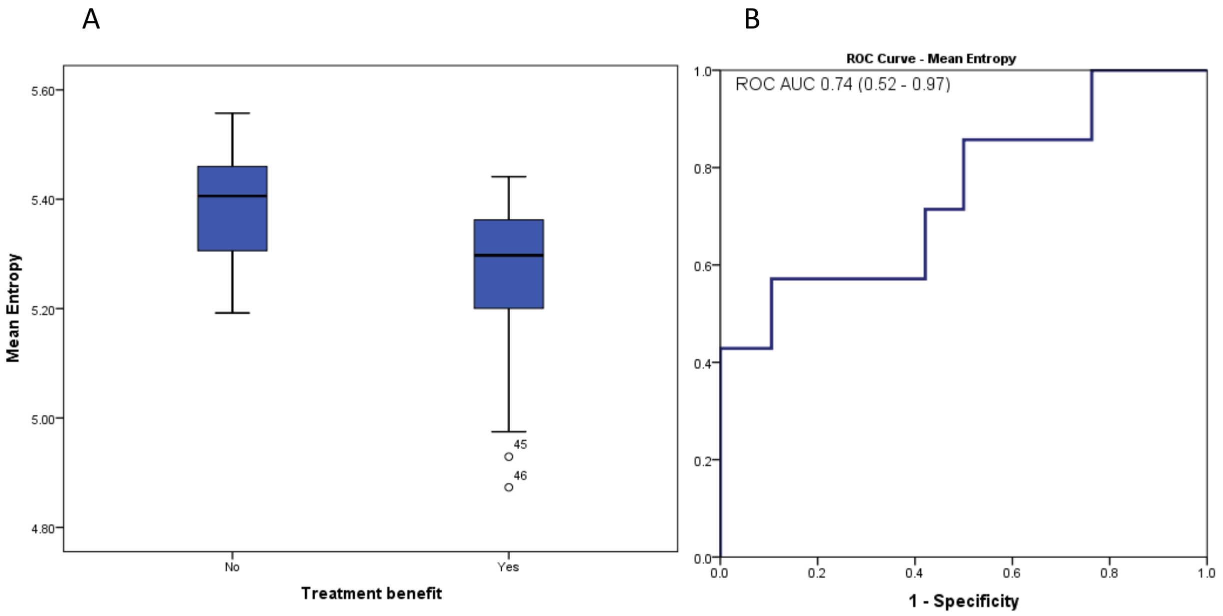

Supplemental figure 1. A: differences in mean Entropy between patients with and without treatment benefit in first-line treatment. **B**: ROC curve of mean Entropy to predict treatment benefit.

First-line treatment group					
	Sidedness				
	Left		Right		p
	Mean	SD	Mean	SD	
Mean SUV_{max}	6.66	2.32	8.34	5.13	0.26
Mean SUV_{peak}	5.16	1.89	6.38	3.50	0.13
Mean SUV_{mean}	4.34	1.41	5.21	2.83	0.17
Mean Compactness	0.04	0.01	0.04	0.01	0.20
Mean Shericity	0.88	0.10	0.83	0.14	0.17
Mean AUC-CSH	0.74	0.03	0.74	0.05	0.50
Mean Entropy	5.30	0.13	5.26	0.17	0.41
Mean Entropy FXD	3.58	0.60	3.74	0.77	0.47
	Sidedness				
	Left		Right		p
	Median	SD	Median	SD	
Mean MATV	12.54	15.93	17.75	64.41	0.05*
SUM MATV	22.57	67.46	68.40	336.70	0.02*
Mean TLG	46.42	62.64	102.75	411.50	0.09
SUM TLG	94.81	290.21	510.86	2031.28	0.02*

Supplemental table 1A. Radiomics versus sidedness of the primary tumour in first-line treatment.

Third-line treatment group										
	Sidedness					RAS / BRAF status				
	Left		Right		p	Wild-type		Mutated		p
	Mean	SD	Mean	SD		Mean	SD	Mean	SD	
Mean SUV_{max}	7.67	2.21	10.01	4.50	0.14	8.41	3.02	8.22	3.05	0.9
Mean SUV_{peak}	6.41	1.77	7.76	3.43	0.10	6.91	2.26	6.55	2.36	0.74
Mean SUV_{mean}	4.84	1.39	5.71	2.02	0.12	5.17	1.55	4.92	1.51	0.74
Mean Compactness	0.03	0.01	0.02	0.01	0.03*	0.03	0.01	0.03	0.01	0.48
Mean Shericity	0.71	0.17	0.56	0.19	0.02*	0.67	0.19	0.61	0.12	0.53
Mean AUC-CSH	0.69	0.10	0.69	0.05	0.80	0.69	0.10	0.69	0.03	0.94
Mean Entropy	5.36	0.17	5.26	0.22	0.14	5.35	0.20	5.24	0.13	0.22
Mean Entropy FXD	3.62	0.62	4.12	0.62	0.03*	3.71	0.66	3.78	0.56	0.83
	Sidedness					RAS / BRAF status				
	Left		Right		p	Wild-type		Mutated		p
	Median	SD	Median	SD		Median	SD	Median	SD	
Mean MATV	32.07	90.59	42.22	104.56	0.43	32.07	103.11	52.54	21.65	0.55
SUM MATV	160.33	274.22	148.35	180.55	0.95	169.15	272.87	195.26	230.48	0.79
Mean TLG	179.48	448.75	188.25	701.47	0.40	188.91	565.48	187.59	224.93	0.79
SUM TLG	789.79	1469.22	817.00	848.73	0.88	841.46	1451.36	789.79	1025.00	0.84

Supplemental table 1B. Radiomics versus sidedness and BRAF/ RAS mutation status of the primary tumour in third-line treatment.

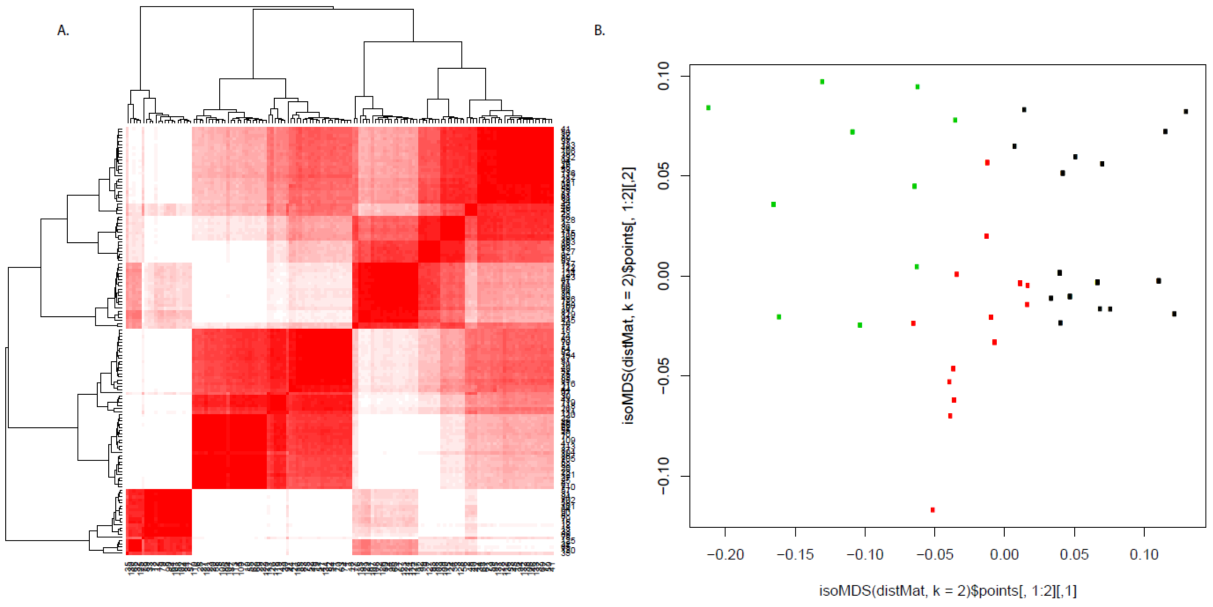

Supplemental figure 2A. A consensus-clustering graph of the first-line treatment group is depicted in A. This graph illustrates the repeatability of clustering if tested in multiple subsets of the main data set. Red and white indicate comparable clustering grouping, pink indicates changes between cluster groups. **B.** Principal component graph of the first-line treatment group. This graph illustrates the correlations between the original data and the cluster data. More clustering of data points per group indicates more significant differences.

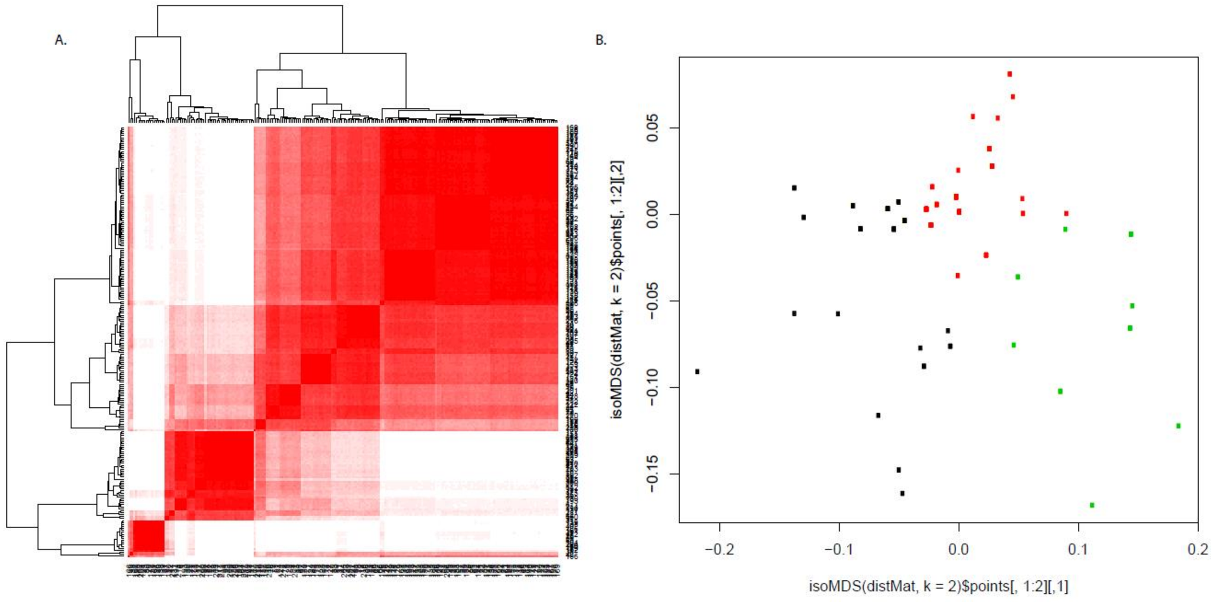

Supplemental figure 3A. Consensus-clustering graph for the third-line treatment group. **B.** Principal component graph of the third-line treatment group.

Correlations among PET characteristics

		SUV_{MAX}	SUV_{PEAK}	SUV_{MEAN}	MATV	TLG	AUC	Compactness	Sphericity	Entropy	Entropy FXD
SUV_{MAX}	Pearson Correlation		.969	.945**	.415**	.641**	-.260**	-.296**	-.311**	.029	.565**
	Sig. (2-tailed)		< 0.001	< 0.001	< 0.001	< 0.001	< 0.001	< 0.001	< 0.001	0.587	< 0.001
	N		354	354	354	354	354	354	354	354	354
SUV_{PEAK}	Pearson Correlation	.969**		.940**	.459**	.677**	-.240**	-.285**	-.302**	.116*	.576**
	Sig. (2-tailed)	< 0.001		< 0.001	< 0.001	< 0.001	< 0.001	< 0.001	< 0.001	0.029	< 0.001
	N	354		354	354	354	354	354	354	354	354
SUV_{MEAN}	Pearson Correlation	.945**	.940**		.299**	.546**	-.101	-.172**	-.184**	.156**	.556**
	Sig. (2-tailed)	< 0.001	< 0.001		< 0.001	< 0.001	0.058	0.001	0.001	0.003	< 0.001
	N	354	354		354	354	354	354	354	354	354
MATV	Spearman Correlation	.415**	.459**	.299**		.948**	-.209**	-.635**	-.635**	.325**	.314**
	Sig. (2-tailed)	.000	.000	.000		<0.001	.000	.000	.000	.000	.000
	N	354	354	354		354	354	354	354	354	354
TLG	Spearman Correlation	.641**	.677**	.546**	.948**		-.203**	-.604**	-.604**	.352**	.472**
	Sig. (2-tailed)	.000	.000	.000	<0.001		.000	.000	.000	.000	.000
	N	354	354	354	354		354	354	354	354	354
AUC	Pearson Correlation	-.260**	-.240**	-.101	-.209**	-.203**		.373**	.382**	.404**	-.014
	Sig. (2-tailed)	< 0.001	< 0.001	0.058	< 0.001	< 0.001		< 0.001	< 0.001	< 0.001	0.786
	N	354	354	354	354	354		354	354	354	354
Compactness	Pearson Correlation	-.296**	-.285**	-.172**	-.635**	-.604**	.373**		.997**	.040	-.006
	Sig. (2-tailed)	< 0.001	< 0.001	0.001	< 0.001	< 0.001	< 0.001		< 0.001	0.453	0.908
	N	354	354	354	354	354	354		354	354	354
Sphericity	Pearson Correlation	-.311**	-.302**	-.184**	-.635**	-.604**	.382**	.997**		.050	-.021
	Sig. (2-tailed)	< 0.001	< 0.001	0.001	< 0.001	< 0.001	< 0.001	< 0.001		.349	.700
	N	354	354	354	354	354	354	354		354	354
Entropy	Pearson Correlation	.029	.116*	.156**	.325**	.352**	.404**	.040	.050		.340**
	Sig. (2-tailed)	0.587	0.029	0.003	< 0.001	< 0.001	< 0.001	.453	.349		< 0.001
	N	354	354	354	354	354	354	354	354		354
Entropy FXD	Pearson Correlation	.565**	.576**	.556**	.314**	.472**	-.014	-.006	-.021	.340**	
	Sig. (2-tailed)	< 0.001	< 0.001	< 0.001	< 0.001	< 0.001	0.786	0.908	0.700	< 0.001	
	N	354	354	354	354	354	354	354	354	354	

**. Correlation is significant at the 0.01 level (2-tailed).

*. Correlation is significant at the 0.05 level (2-tailed).

Supplemental table 2. This table depicts the correlations among PET characteristics. Red indicate the absence of a correlation.

Supplemenatry data 1

In the first-line treatment group, there were no differences in SUV_{max} (p=0.4), SUV_{peak} (p=0.6), SUV_{mean} (p=0.45), MATV (p=0.38), TLG (0.56) or Entropy (0.51) and different sites of metastases. There were significant differences between organ sites of metastases and Compactness (p=0.03), Sphericity (p=0.03) and AUC (p=0.04).

First-line treatment cohort

	SUVmax		SUVpeak		SUVmean		MATV		TLG		Compactness		Sphericity		AUC-CSH		Entropy		Entropy FXD	
	Mean	Standard Deviation	Mean	Standard Deviation	Mean	Standard Deviation	Median	Standard Deviation	Median	Standard Deviation	Mean	Standard Deviation	Mean	Standard Deviation	Mean	Standard Deviation	Mean	Standard Deviation	Mean	Standard Deviation
Colon	8,59	3,70	6,59	2,98	5,31	2,19	10,88	15,76	52,65	128,11	,04	,01	,83	,15	,72	,05	5,32	,16	3,98	,63
Peritoneal	6,14	2,21	4,84	1,65	3,84	1,28	7,55	56,76	30,87	198,24	,04	,01	,80	,18	,71	,05	5,21	,32	3,50	,65
Lungs	5,92	1,38	4,80	1,19	3,78	,90	14,46	4,89	52,55	5,53	,04	,00	,82	,06	,74	,01	5,51	,06	3,53	,29
Liver	6,77	3,29	5,49	2,61	4,48	1,94	7,97	147,14	36,71	902,89	,05	,01	,90	,17	,75	,06	5,29	,21	3,52	,73
Adrenalgland	5,16	,26	4,14	,08	3,46	,14	21,34	18,87	72,60	62,36	,04	,00	,82	,06	,69	,05	5,37	,19	3,36	,14
Bone	6,07	3,13	4,93	2,47	3,84	1,90	15,62	23,22	108,32	67,10	,04	,01	,80	,14	,72	,04	5,38	,14	3,39	,80
Lymphnode	6,29	1,68	4,61	1,02	3,97	1,05	8,13	7,06	31,94	29,41	,04	,01	,79	,16	,74	,04	5,26	,21	3,60	,48
Spleen	6,19	2,91	4,03	,74	4,04	1,77	12,83	11,99	41,20	25,72	,04	,01	,88	,12	,77	,01	5,19	,25	3,59	1,00
Other	5,79	,23	4,60	,44	4,10	,16	12,16	3,44	48,57	16,01	,05	,00	,93	,05	,73	,05	5,45	,18	3,76	,30

Post Hoc analysis, First-line treatment group

	Compactness (p-value)	Sphericity (p-value)	AUC-CSH (p-value)
Liver versus Colon	**0.02**	**0.02**	**<0.01**
Liver versus Peritoneal	**0.02**	**0.02**	**0.01**
Liver versus Lungs	0.24	0.24	0.51
Liver versus Adrenal Gland	0.24	0.24	0.17
Liver versus Bone	0.10	0.10	0.09
Liver versus Lymph node	**0.01**	**0.01**	0.21
Liver versus Spleen	0.61	0.61	0.91
Liver versus Other	0.70	0.70	0.38

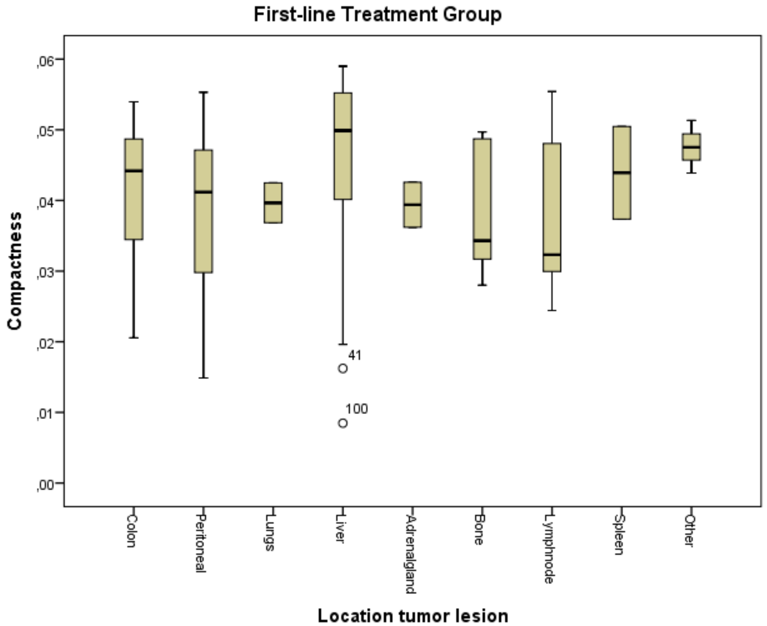
First-line Treatment Group
Compactness
,06
,05
,04
,03
,02
,01
,00
41
100
Colon
Peritoneal
Lungs
Liver
Adrenalgland
Bone
Lymphnode
Spleen
Other
Location tumor lesion

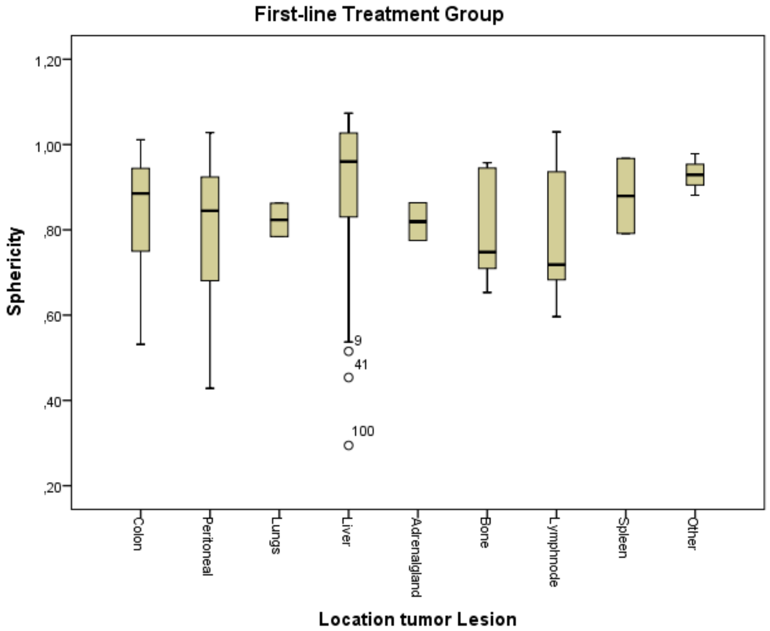
First-line Treatment Group
Sphericity
1,20
1,00
,80
,60
,40
,20
9
41
100
Colon
Peritoneal
Lungs
Liver
Adrenalgland
Bone
Lymphnode
Spleen
Other
Location tumor Lesion

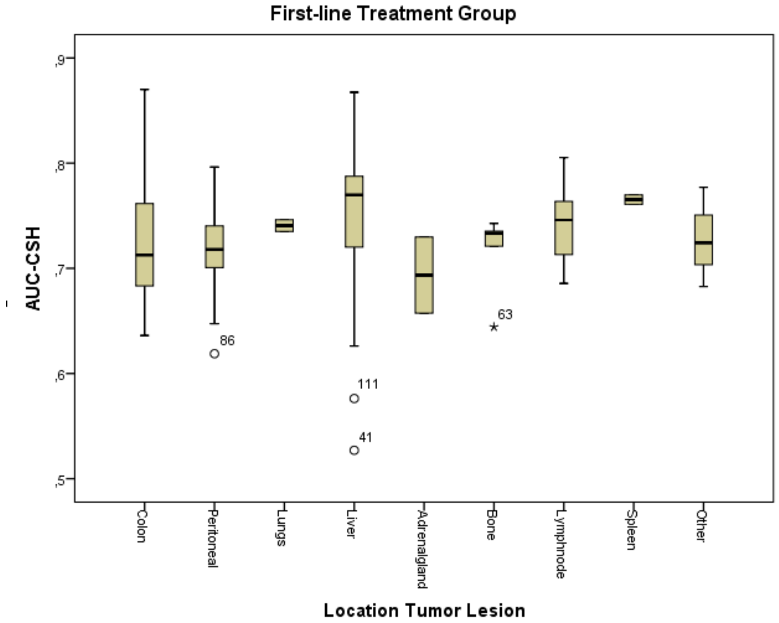
First-line Treatment Group
AUC-CSH
,9
,8
,7
,6
,5
86
111
41
63
Colon
Peritoneal
Lungs
Liver
Adrenalgland
Bone
Lymphnode
Spleen
Other
Location Tumor Lesion

In third-line treatment group, there were no differences in MATV (p=41), TLG (p=0.20), Compactness (p=0.22), Sphericity (p=0.22), or AUC (p=0.44) and different sites of metastases. There were significant differences between organ sites of metastases and SUV_{max} (p<0.01), SUV_{peak} (p<0.01), SUV_{mean}, (p<0.01), and Entropy (p<0.01).

Third-line treatment cohort

	SUVmax		SUVpeak		SUVmean		MATV		TLG		Compactness		Sphericity		AUC-CSH		Entropy		Entropy FXD	
	Mean	Standard Deviation	Mean	Standard Deviation	Mean	Standard Deviation	Median	Standard Deviation	Median	Standard Deviation	Mean	Standard Deviation	Mean	Standard Deviation	Mean	Standard Deviation	Mean	Standard Deviation	Mean	Standard Deviation
Colon	8,80	3,26	7,12	2,88	5,52	2,02	15,55	22,54	66,46	210,37	,03	,01	,74	,21	,75	,06	5,44	,14	3,86	1,20
Peritoneal	9,85	5,54	7,61	4,29	5,55	2,52	10,75	60,07	47,71	640,03	,02	,01	,56	,18	,65	,23	5,14	,38	2,60	1,97
Lungs	6,57	2,11	5,27	1,82	4,18	1,27	10,43	86,15	42,03	460,66	,04	,02	,76	,23	,67	,10	5,07	,40	3,42	,67
Liver	7,99	2,61	6,67	2,14	5,13	1,66	19,26	136,05	98,91	723,64	,03	,01	,72	,21	,69	,13	5,36	,28	3,71	,78
Adrenalgland	8,26	3,15	6,42	2,22	5,17	1,53	20,78	19,39	93,89	127,73	,03	,01	,73	,12	,73	,06	5,48	,17	3,85	,89
Bone	5,69	1,26	4,69	1,05	3,58	,60	36,48	56,08	147,56	245,94	,03	,01	,65	,12	,65	,18	5,21	,43	3,16	,49
Lymphnode	7,01	2,97	5,52	2,24	4,29	1,71	10,88	52,25	55,57	182,18	,03	,01	,66	,16	,69	,12	5,31	,27	3,37	,94
Soft tissue	11,41	2,60	8,97	2,09	6,89	1,69	19,01	32,10	81,19	227,05	,03	,02	,66	,30	,67	,05	5,26	,48	4,21	1,74

Post Hoc analysis, Third-line treatment group

	SUV_{max} (p-value)	SUV_{peak} (p-value)	SUV_{mean} (p-value)	Entropy (p-value)
Liver versus Colon	0.60	0.86	0.72	0.49
Liver versus Peritoneal	0.59	0.88	0.86	0.10
Liver versus Lungs	**0.02**	**0.011**	**0.02**	**<0.01**
Liver versus Adrenal Gland	0.91	0.62	0.93	0.30
Liver versus Bone	**<0.01**	**<0.01**	**<0.01**	0.36
Liver versus Lymph node	**<0.01**	**<0.01**	**<0.01**	0.30
Liver versus Other	**<0.01**	**0.02**	**0.03**	0.79

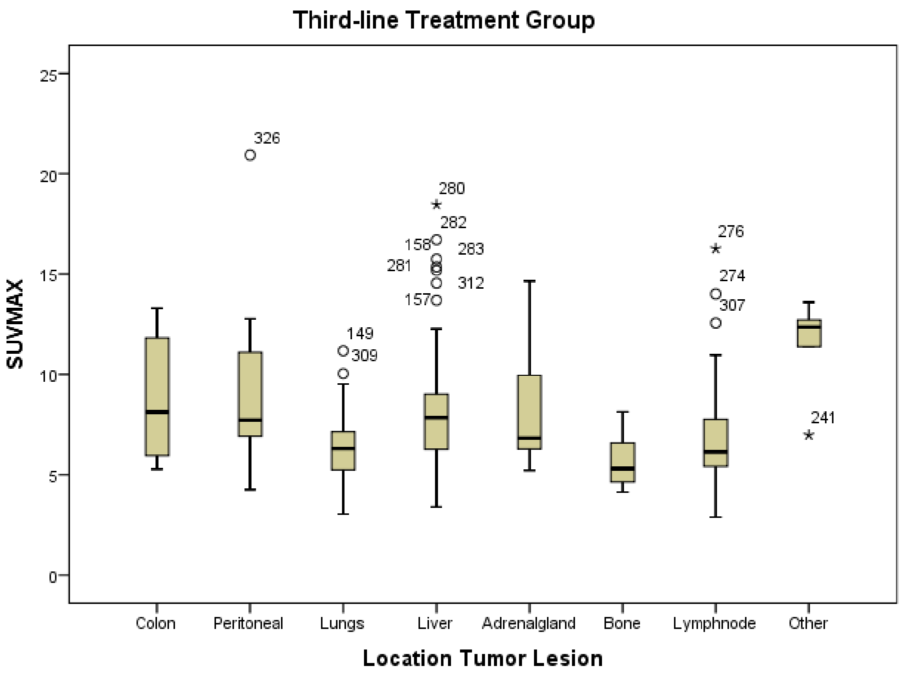
Third-line Treatment Group
SUVMAX
0
5
10
15
20
25
326
280
282
158
283
281
312
157
149
309
276
274
307
241
Colon
Peritoneal
Lungs
Liver
Adrenalgland
Bone
Lymphnode
Other
Location Tumor Lesion

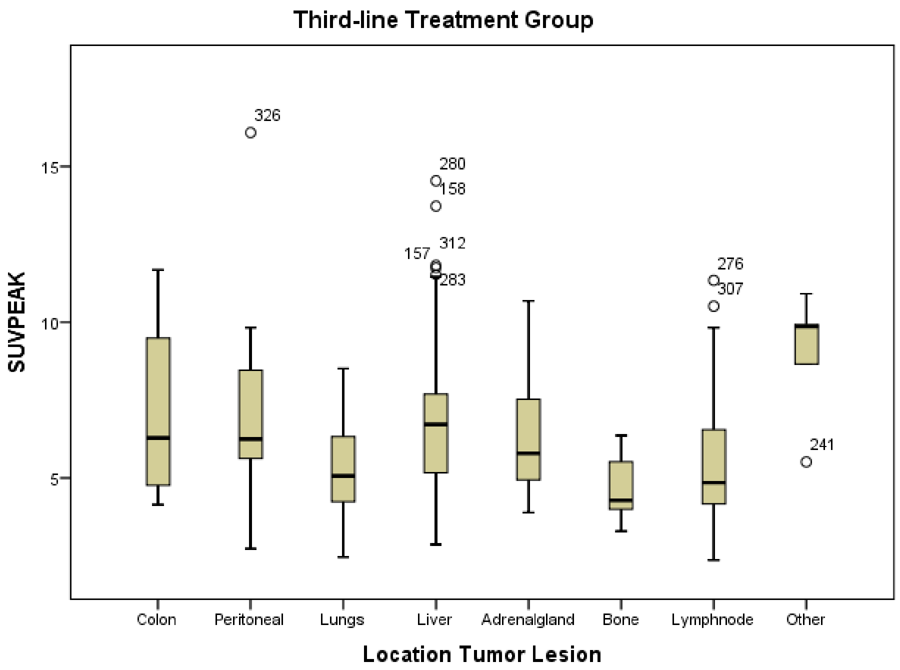
Third-line Treatment Group
SUVPEAK
5
10
15
326
280
158
157
312
283
276
307
241
Colon
Peritoneal
Lungs
Liver
Adrenalgland
Bone
Lymphnode
Other
Location Tumor Lesion

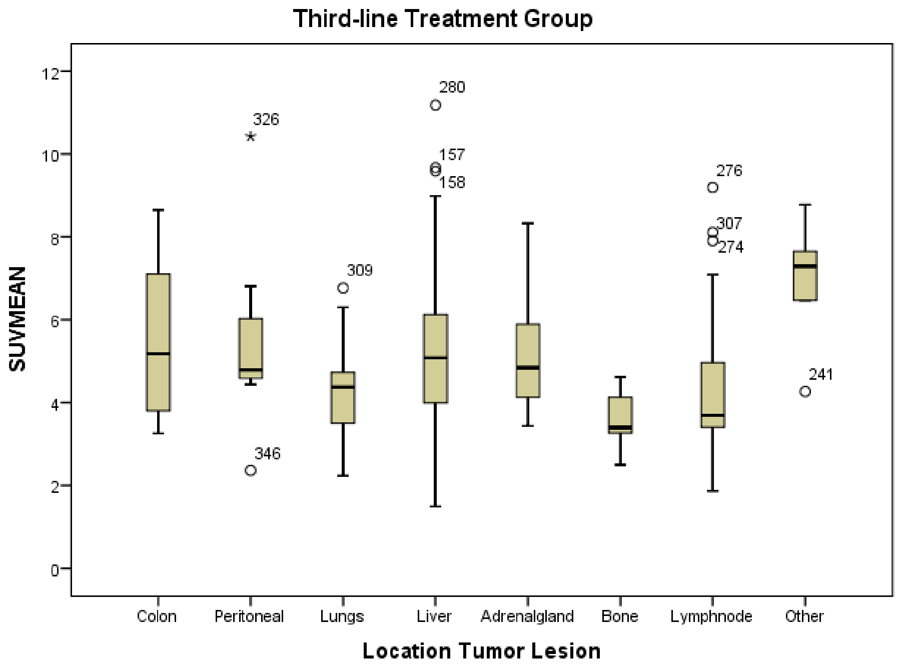
Third-line Treatment Group
SUVMEAN
0
2
4
6
8
10
12
326
346
280
157
158
309
276
307
274
241
Colon
Peritoneal
Lungs
Liver
Adrenalgland
Bone
Lymphnode
Other
Location Tumor Lesion

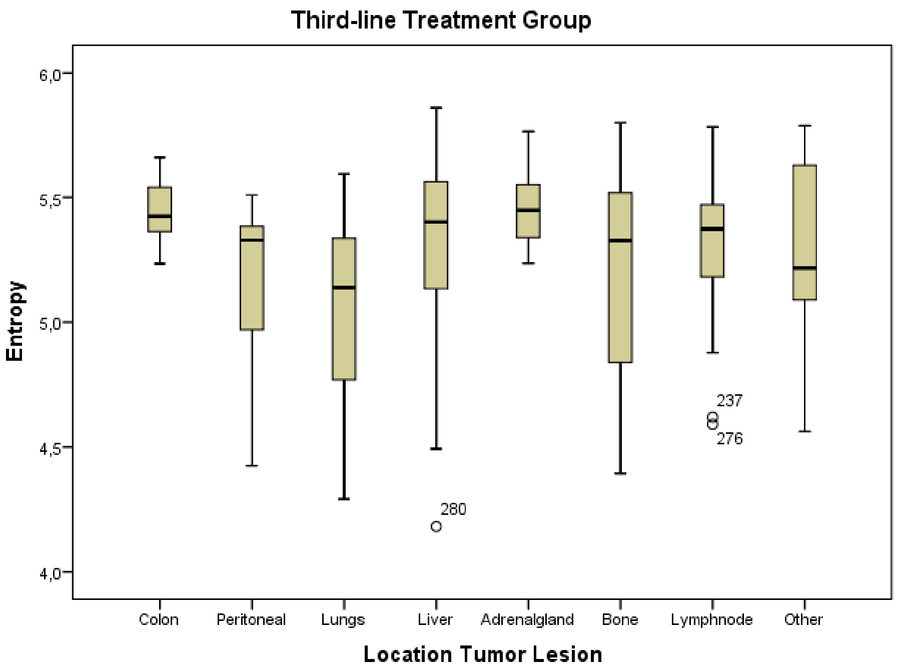
Third-line Treatment Group
Entropy
4,0
4,5
5,0
5,5
6,0
280
237
276
Colon
Peritoneal
Lungs
Liver
Adrenalgland
Bone
Lymphnode
Other
Location Tumor Lesion
